# Post-traumatic Stress and Growth Among the Children and Adolescents in the Aftermath of COVID-19

**DOI:** 10.3389/fpsyg.2021.791263

**Published:** 2022-01-27

**Authors:** Braj Bhushan, Sabnam Basu, Umer Jon Ganai

**Affiliations:** Department of Humanities and Social Sciences, Indian Institute of Technology Kanpur, Kanpur, India

**Keywords:** COVID-19, post-traumatic stress (PTS), post-traumatic growth, children and adolescents, direct-indirect media exposure

## Abstract

The coronavirus disease 2019 (COVID-19) pandemic has enkindled many mental health problems across the globe. Prominent among them is the prevalence of post-traumatic stress (PTS) with hosts of its precipitating factors being present in the surrounding. With India witnessing severe impact of the second wave of COVID-19, marked by a large number of hospitalizations, deaths, unemployment, imposition of lockdowns, etc., its repercussions on children and adolescents demand particular attention. This study aims to examine the direct and the indirect exposure of COVID-19-related experiences on children and adolescents and its subsequent relationship with PTS and post-traumatic growth (PTG). The direct exposure was operationalized in terms of death or hospitalization in the family, while the indirect exposure was gauged in terms of exposure to media reports of the COVID situation. Data from 412 children and adolescents aged 9–20 years, collected online, revealed 68.9% of them with PTS. Interestingly, 39.8% of those reporting PTS were also experiencing PTG. Arousal appeared to be the most frequently reported characteristics of trauma. The multivariate analysis of variance (MANOVA) endorses significant difference between those with direct and indirect exposures to hospitalization. Those with direct exposure to hospitalization reported higher PTS. The indirect exposure of COVID-19-related news through electronic media was also significantly associated with higher PTS. Exposure through print media did not lead to significant difference in PTS, but those reading only magazines reported significantly higher PTG than not reading magazines. The findings are analyzed in the light of unfolding of events during the second wave of COVID-19 in India.

## Introduction

What began as common flu-type symptoms in December 2019 soon overtook the world, spreading at an exponential rate. By (World Health Organization [WHO], 2020) declared coronavirus disease 2019 (COVID-19) outbreak a pandemic. To control the rapid escalation of COVID-19, nations across the world tried to adopt various measures, including lockdown, which lasted from several weeks to several months. India also adopted this strategy to control the spread of the virus, announcing a nationwide lockdown on 24 March 2020 that was further extended till 3 May 2020 (Ministry of Home Affairs, 2020), impacting the daily lives of people drastically. Despite such efforts, the scenario became worse with the second wave of COVID that hit the country in March 2021. This was much more severe than the first wave, both in terms of symptom escalation and fatality rate, as well as shortage of hospital beds, oxygen supply, and medicines in parts of the country (Safi, 2021). By late April, India became the country with the highest number of new and active cases (BBC, 2021). The policy of test, trace, and treat, and emphasis on COVID-19 specific behavior, along with vaccination resulted in the decline in positive cases during the first wave (Joshi and Mehendale, 2021). However, these measures could not curtail the spread of the virus in the community and resulted in the worst outbreak of the second wave. To add to this turmoil, many cases of mucormycosis (black fungus) and aspergillosis (white fungus) started surfacing. During the first wave, people infected with COVID were predominantly older than 60 years. However, the second wave claimed the lives of people aged 25–50 years (Jain et al., 2021).

The existing research in psychological science leaves us with a conjecture of likelihood of mental health-related issues in the aftermath of the pandemic, especially in those who have been infected. Disabling conditions such as post-traumatic stress (PTS) are prevalent among people because of traumatic events such as infectious diseases. Joseph and Williams (2005) have argued that “the phenomena of distressing intrusive thoughts, images, and pictures accompanied by the need to avoid these distressing emotions, thoughts, and reminders. To be normal reactions experienced by people in response to stressful and traumatic situations, indicative of the need for cognitive-emotional processing. Rather than an abnormal state of mind” (p. 426). Accordingly, several researchers have preferred to use the term “post-traumatic stress” instead of “post-traumatic stress disorder” (Bhushan and Kumar, 2007, 2009, 2012). We reiterate the same and have used the term “PTS” here. In a systematic review of the literature, Vindegaard and Benros (2020) have reported PTS in individuals exposed to COVID-19. They reviewed 43 studies. Two of them examined 919 adults with COVID-19 and found high prevalence (96.2%) of PTS. The remaining forty-one studies (2 on patients with preexisting psychiatric disorders, 20 on medical healthcare workers, and 19 on the general public) evaluated the indirect effect of the pandemic. There is an obvious absence of children and adolescents in these studies. Very few studies have focused on this vulnerable segment. Chen et al. (2021) studied the prevalence of PTS among 15,993 children and adolescents aged 8–18 years in the aftermath of COVID-19 in China. A total of 11.5% of participants met the clinical criteria. Selçuk et al. (2021) examined 447 adolescents during the COVID-19 outbreak on the moderate prevalence level of 28.5% of PTS in Turkey. This necessitates research focusing on children and adolescents. Given the likelihood of PTS in the wake of tremendously distressful situations such as COVID-19, high rates of PTS-like symptoms such as intrusion, avoidance of stimuli, negative alterations in cognition or mood, and marked alterations in arousal and reactivity have been reported in the survivors (Xiao et al., 2020). A recent review of 19 studies consisting a total of 93,569 participants shows the prevalence of PTS ranging from 7 to 53.8% in the general population (Xiong et al., 2020).

The pandemic has also affected the mental health of children and adolescents (Golberstein et al., 2020). Studies suggest that continued closures of academic institutions, isolation from peers and others in the social network, and economic closure have severely affected the mental health of children and adolescents (Holmes et al., 2020; Tan et al., 2020). Sleep disorders and PTS are the most severe among the mental health problems, and about half of the children examined experienced them during the pandemic. The systematic review and meta-analysis by Ma et al. (2021), consisting of 23 studies with a total of 57,927 children and adolescents aged 0–18 years, provide evidence for depression in 28.6%, anxiety in 25.5%, sleep disorders in 44.2%, and PTS in 48.0% of children and adolescents during COVID-19. Hyland et al. (2021) examined depression and generalized anxiety disorder (GAD) during 6 weeks of lockdown in Ireland and found that the prevalence of neither depression nor GAD of affected during this phase of COVID-19. However, younger age was found to predict major depression.

Pre-COVID literature suggests an association between media exposure to traumatic events and PTS, ranging from high acute stress after media exposure to the Boston Marathon bombings (Holmes et al., 2020) to indirect exposure to the 2008 Wenchuan earthquake (Yeung et al., 2018). Pfefferbaum et al. (2001) found a positive correlation between viewing time of Oklahoma City bombing-related news and PTS symptoms in children who were unaffected by the incidence. Similar correlation between the duration of television exposure to scarred graphic images and PTS severity has been reported in Kuwaiti children and adolescents after the military occupation (Nader et al., 1993). Bhushan and Kumar (2009) compared PTS in children directly exposed to tsunami in India and those who were exposed to it through media and concluded that a traumatic event could result in PTS even in children who get indirectly exposed to it through media. They observed a significant sex difference on PTS, with women at a higher risk compared with men. Recent studies have also associated viewing COVID-19-related news even at the time of the outbreak with negative affect, anxiety, depression, and stress (Chao et al., 2020) and heightened fear of the virus after searching further information about the pandemic through different mediums (Mertens et al., 2020). The Director-General of WHO even went ahead referring to “infodemic” drawing a resemblance between the spread of news and the virus (Zarocostas, 2020).

A parallel body of research suggests positive psychological changes after the traumatic experiences, termed as post-traumatic growth (PTG, Tedeschi and Calhoun, 1995). PTG involves increased sense of personal strength, feeling of connectedness with others, finding new possibilities and opportunities, greater appreciation for life, and a deeper understanding of spiritual and existential questions (Tedeschi and Calhoun, 1996). Calhoun and Tedeschi (2006) explained PTSD and PTG as independent psychological constructs where the cognitive process activated by the distress experienced by the survivors result in a positive outlook of the self, others, and the world, resulting into PTG. This implies co-occurrence of PTSD and PTG. Recent studies have reported the presence of both negative psychological reactions and PTG during the COVID pandemic (Tomaszek and Muchacka-Cymerman, 2020; Cui et al., 2021; Hamam et al., 2021). Cui et al. (2021) found the prevalence of positive psychological reactions in 43.7% of nurses in China who reported above average PTG scores. Children and adolescents, the two most vulnerable groups, are underrepresented in the available studies related to COVID. This was the motivating factor behind undertaking this study.

### The Present Study

This study aims to examine the direct and the indirect exposure of COVID-19-related experiences on children and adolescents and its subsequent relationship with PTS and PTG. It examines the prevalence of PTS and PTG among children and adolescents in India during the second wave of COVID-19. Furthermore, we investigated the difference between those with adversities at home arising out of COVID and those who were exposed to it through electronic and print media in terms of PTS. Earlier studies of PTS in tsunami-affected children and adolescents of India (Bhushan and Kumar, 2007, 2009) have shown higher level of trauma in this vulnerable group. Even 30.69–37.62% of those who were indirectly exposed to tsunami had relatively high intrusion (Bhushan and Kumar, 2009). In the absence of any study reporting the prevalence of PTS in children and adolescents of India during the COVID-19 and a single study reporting a prevalence of 28.2% in adults (Singh and Khokhar, 2021), we hypothesized (i) relatively high prevalence of PTS and PTG among the children and adolescents, (ii) significant difference in PTS (intrusion, avoidance, and arousal) among children and adolescents witnessing hospitalization compared with those who did not, (iii) significant difference in PTS (intrusion, avoidance, and arousal) among children and adolescents who witnessed death in family compared with those who did not, and (iv) significant difference in PTS (intrusion, avoidance, and arousal) among children and adolescents who have had electronic and/or print media exposure as compared with those who did not. We also explored the coexistence of PTS and PTG in children and adolescents who reported PTS during the second wave of COVID.

## Materials and Methods

### Participants

The sample comprised of 412 children and adolescents (226 men and 186 women) aged 9–20 years (*M* = 15.12, *SD* = 2.15). Following the conventional practice in psychological science, we have operationally defined children as those belonging to the age range of 9–11 years and adolescents as those belonging to the age range of 12–20 years. Accordingly, 3.7% of the participants were children, while the remaining 96.3% were adolescents. Almost half of the participants (43%) belonged to the urban areas, while the remaining (57%) belonged to the rural areas. Of the participants, 89% had one or more siblings and half of them lived along with their grandparents (50%). Age ranging between 9 and 20 years with no reported symptoms of psychological distress or clinical prognosis was the inclusion criteria. Ongoing hospitalization of any close family member or diagnosed psychological problem was the exclusion criteria. None of the participants had COVID-19.

### Measures

Information was collected about the demographics of the participants (e.g., age, gender, family size, rural or urban, and family type). The participants also reported hospitalization and death in the family due to the pandemic and exposure to the electronic and print media in a binary (“Yes” or “No”) format.

The Children’s Revised Impact of Event Scale (CRIES-13), a 13-item scale adapted from the Impact of Event Scale (IES, Horowitz et al., 1979; Perrin et al., 2005), was administered to the participants to identify the likelihood of PTS associated with the COVID-19 pandemic. The items of CRIES are rated on a four-point scale, according to the frequency of recurrence of the PTS reactions during the week before administration of the tool. The total score ranges between 0 and 65 and is attained from the scores of the three subscales (intrusion, avoidance, and arousal). Researchers have suggested that a cutoff score of 17 or above on the intrusion, avoidance, and arousal scale correctly identifies more than 80% of children diagnosed with PTS (Yule, 1998; Stallard et al., 1999). A cutoff of 30 indicates children at risk for PTS and has been known to maximize the sensitivity and specificity while minimizing the rate of false negatives (Perrin et al., 2005). The entire scale has a good internal consistency (Cronbach’s alpha 0.78), while the subscales individually have acceptable reliabilities (avoidance = 0.613; intrusion = 0.60; arousal = 0.612).

The Revised Post-traumatic Growth Inventory for Children (PTGI-C-R, Kilmer et al., 2009) was administered to measure PTG. Adapted from the PTGI, 10 out of the 21 original items have been selected for the PTGI-C-R, considering their suitability to children. The participants responded to these items on a 4-point rating scale (ranging from 0, no change, to 3, a lot of change including a “don’t know” option). Cronbach’s alpha for the total scale in this study is 0.70.

### Procedure

India was badly hit by the second wave of COVID-19 between March and May 2021. The data were collected using Google survey forms in July 2021, 30 days after the second wave had lost its impetus. The link for the survey forms was circulated among the parents/guardians through specific e-groups of students and/or parents created after the schools were closed due to COVID. They were also contacted through their respective schools, and those who consented were asked to respond to the demographic profile form and the two tools. A total of 422 participants were involved through snowball sampling. The participants, as well as their parents, were informed about the voluntary nature of participation and their right to withdraw for any reason at any time during the data collection period. All this information were included in the informed consent form provided at the beginning of the survey form. The completion of the entire survey took approximately 8–10 min. As these children and adolescents were already involved in online classes conducted by their respective schools, they were familiar with online modes, such as using smartphones, personal computers, or laptops. The study protocol was approved by the Institutional Ethics Committee of the Indian Institute of Technology Kanpur. A total of 422 responses were obtained, out of which 412 were eligible for further analysis. Ten responses were rejected as they were incomplete.

### Analysis

Besides descriptive statistics, the multivariate analysis of variance (MANOVA) was performed to analyze the differences in PTS (intrusion, avoidance, and arousal) and PTG scores among children and adolescents with different levels of exposure to COVID-19. All the statistical analysis was carried out using SPSS version 21. An important assumption for carrying out MANOVA is that all the dependent variables should have a moderate correlation with each other (Meyers et al., 2016). Thus, the Pearson correlation among all the dependent variables was calculated.

## Results

### Descriptive and Correlational Statistics

As summarized in Table 1, all the dependent variables were significantly positively correlated to each other. The highest correlation coefficient was observed between PTS and its three components, namely, intrusion (*r* = 0.799; *p* < 0.01), avoidance (*r* = 0.746; *p* < 0.01), and arousal (*r* = 0.832; *p* < 0.01). PTS and PTG were positively correlated (*r* = 0.281; *p* < 0.01) albeit the coefficient was low.

**TABLE 1:** Mean, standard deviation (SD), and correlation among all the variables of the study.

Variables	Mean	SD	1	2	3	4	5
1. Intrusion	9.75	4.42	1				
2. Avoidance	10.62	4.77	0.410**	1			
3. Arousal	12.82	5.49	0.536**	0.387**	1		
4. PTS	33.19	11.66	0.799**	0.746**	0.832**	1	
5. PTG	19.22	4.96	0.275**	0.247**	0.161**	0.281**	1

**p < 0.01.

### Occurrence of Post-traumatic Stress and Post-traumatic Growth

Table 2 presents the prevalence of PTS and PTG in the present sample. Keeping 17 as the cutoff for the subscales and 30 as the cutoff for the entire scale (as cited above), we found 284 out of the 412 participants to be affected by PTS, leading to a prevalence rate of 68.9%. Arousal emerged to be the most frequently associated characteristics of trauma (31.6%). A total of 113 children and adolescents suffering from PTS were also found to report PTG, accounting for 39.79% prevalence of PTG among those experiencing PTS. Thus, the first hypothesis claiming relatively high prevalence of PTS and PTG among the children and adolescents was accepted.

**TABLE 2:** Prevalence of post-traumatic stress (PTS) and post-traumatic growth (PTG) in the present sample.

	N	Frequency	Percent
Intrusion	412	10	2.4%
Avoidance	412	35	8.5%
Arousal	412	130	31.6%
Post-traumatic stress	412	284	68.9%
PTG among participants suffering from PTS	284	113	39.79%

### Multivariate Analysis of Variance

Table 3 demonstrates the groupwise difference for the demographic variables such as age, gender, location, and family on intrusion, avoidance, arousal, PTS, and PTG. The MANOVA suggests that of all the demographic variables, only location emerged to have a significant effect [Wilks’ λ = 0.94, *F*_(4, 368)_ = 6.196, *p* < 0.001]. Pairwise comparison revealed that participants living in rural areas experienced significantly higher intrusion and PTS scores as compared with those living in urban areas. Although other demographic variables did not yield significant outcome in multivariate testing, univariate testing showed that participants who had at least one or more siblings experienced higher PTG as compared with those without siblings.

**TABLE 3:** Multivariate analysis of variance (MANOVA) table reporting the group difference for the demographic variables on intrusion, avoidance, arousal, PTS, and PTG.

	Age		*p*-value	Gender		*p*-value	Location		*p*-value	Siblings		*p*-value	Grandparents		*p*-value
	Children (*N* = 15)	Adolescents (*N* = 376)		Males (*N* = 212)	Females (*N* = 179)		Urban (*N* = 169)	Rural (*N* = 222)		Yes (*N* = 348)	No (*N* = 43)		Yes (*N* = 196)	No (*N* = 195)	
Intrusion	9.84 (1.205)	9.38 (0.41)	0.718	10.34 (0.69)	8.74 (0.72)	0.109	7.47 (0.56)	12.08 (0.87)	<0.001	10.23 (0.49)	8.27 (1.09)	0.102	9.74 (0.52)	9.41 (0.75)	0.717
Avoidance	11.29 (1.32)	9.33 (0.44)	0.162	10.24 (0.76)	9.79 (0.78)	0.680	10.27 (0.62)	9.71 (0.95)	0.618	10.61 (0.54)	8.91 (1.19)	0.193	9.71 (0.57)	10.22 (0.83)	0.605
Arousal	11.21 (1.54)	12.56 (0.52)	0.408	12.44 (0.88)	11.75 (0.92)	0.588	11.01 (0.72)	13.41 (1.11)	0.070	12.36 (0.63)	11.59 (1.39)	0.614	11.94 (0.66)	12.20 (0.97)	0.825
PTS	32.34 (3.15)	31.28 (1.06)	0.750	33.02 (1.81)	30.28 (1.87)	0.293	28.76 (1.47)	35.19 (2.26)	0.018	33.20 (1.29)	28.77 (2.84)	0.157	31.39 (1.35)	31.83 (1.97)	0.852
PTG	20.20 (1.36)	18.79 (0.46)	0.324	19.09 (0.78)	19.47 (0.81)	0.733	18.84 (0.63)	19.82 (0.97)	0.402	20.31 (0.56)	17.37 (1.22)	0.029	20.45 (0.58)	18.50 (0.85)	0.059
Multivariate test (Wilks’ λ)	0.988, *F*_(4, 368)_ = 1.159		0.329	0.991, *F*_(4, 368)_ = 0.812		0.518	0.937, *F*_(4, 368)_ = 6.196		<0.001	0.982, *F*_(4, 368)_ = 1.643		0.163	0.988, *F*_(4, 368)_ = 1.122		0.346

Table 4 enumerates the results of the MANOVA testing the difference between participants who witnessed hospitalization and/or death in the family during the second wave of COVID-19 in India and those who did not. A statistically significant MANOVA was obtained for hospitalization [Wilks’ λ = 0.95, *F*_(4, 405)_ = 5.404, *p* < 0.001] but not for death [Wilks’ λ = 0.991, *F*_(4, 405)_ = 0.905, *p* = 0.461]. Pairwise comparisons for intrusion, avoidance, arousal, and the overall PTS score show significant difference along hospitalization and no hospitalization. Hence, the second hypothesis was also accepted. The participants who witnessed hospitalization recorded high on intrusion, avoidance, arousal, and PTS. As for PTG, no significant difference was observed between the two groups. As the participants who witnessed the death in the family due to the COVID-19 pandemic did not differ on PTS than those who had no deaths in family, the third hypothesis was rejected.

**TABLE 4:** MANOVA table reporting the group difference based on direct exposure to trauma in the form of hospitalization and death.

	Hospitalization		*p*-value	Death		*p*-value
	Yes (*N* = 127)	No (*N* = 285)		Yes (*N* = 21)	No (*N* = 391)	
Intrusion	12.17 (0.59)	8.59 (0.88)	0.001	10.65 (1.03)	10.11 (0.24)	0.612
Avoidance	12.69 (0.63)	8.27 (0.95)	<0.001	9.83 (1.11)	11.12 (0.26)	0.259
Arousal	16.11 (0.71)	11.74 (1.06)	0.001	14.27 (1.24)	13.59 (0.29)	0.593
PTS	40.97 (1.49)	28.60 (2.24)	<0.001	34.75 (2.62)	34.82 (0.61)	0.979
PTG	19.59 (0.68)	18.06 (1.02)	0.214	18.18 (1.20)	19.47 (0.28)	0.296
Multivariate test (Wilks’ λ)	0.949, *F*_(4, 405)_ = 5.404		<0.001	0.991, *F*_(4, 405)_ = 0.905		0.461

Table 5 enumerates the result of MANOVA testing the difference between participants with massive exposure to news through electronic media as compared with those who did not and participants who read COVID-related news through the print media (newspaper and/or magazines) compared with those who did not. MANOVA results show a statistically significant effect for the electronic media [Wilks’ λ = 0.908, *F*_(4, 405)_ = 10.227, *p* < 0.001] but not for the print media [Wilks’ λ = 0.984, *F*_(4, 405)_ = 1.617, *p* = 0.169]. Pairwise comparisons show significant difference between participants who watched news on electronic media and those who did not in terms of intrusion, avoidance, arousal, overall PTS as well as PTG with exposed group having higher scores on all these parameters. Although the MANOVA result did not emerge to be statistically significant for the print media, univariate testing revealed that participants who relied on print media during this stressful period had statistically higher intrusion scores as compared with those who did not read newspapers and/or magazines. As we only found evidence of difference in PTS (intrusion, avoidance, and arousal) for those who watched electronic media and those who did not, and not for print media, the fourth hypothesis is partially accepted.

**TABLE 5:** MANOVA table reporting the group difference based on indirect exposure to trauma in the form of news through electronic media and print media.

	Electronic media		*p*-value	Print media		*p*-value
	Yes (*N* = 302)	No (*N* = 110)		Yes (*N* = 310)	No (*N* = 102)	
Intrusion	10.31 (0.34)	7.40 (0.40)	<0.001	9.40 (0.32)	8.32 (0.42)	0.039
Avoidance	10.91 (0.38)	8.47 (0.44)	<0.001	9.78 (0.35)	9.60 (0.46)	0.741
Arousal	13.12 (0.43)	10.38 (0.50)	<0.001	12.37 (0.40)	11.12 (0.53)	0.061
PTS	34.34 (0.89)	26.24 (1.04)	<0.001	31.56 (0.83)	29.03 (1.09)	0.065
PTG	19.63 (0.40)	17.61 (0.47)	0.001	19.01 (0.37)	18.24 (0.49)	0.212
Multivariate test (Wilks’λ)	0.908, *F*_(4, 405)_ = 10.227		<0.001	0.984, *F*_(4, 405)_ = 1.617		0.169

## Discussion

This study aims to examine the prevalence of PTS among the children and adolescents of India during the second wave of COVID-19, the subsequent prevalence of PTG in those experiencing PTS, and lastly, to explore the effect of the direct (hospitalization and death) and indirect exposure (electronic and print media) of COVID-19 situation on PTS and PTG of the children and adolescents.

The findings of this study endorse that children and adolescents in India exhibited PTS as highlighted by a prevalence rate of 68.9%, with arousal (31.6%) being the most frequently reported symptom. The findings of this study show 39.79% of the children and adolescents with PTS also experience PTG. This unequivocally suggests coexistence of the two, at least for some time. A wide range of prevalence for PTS has been reported in literature concerning COVID-19. Hou et al. (2020) studied the prevalence of PTS in adolescents and children in China in the aftermath of COVID-19 and found a prevalence rate of 85.5%, while Zhang et al. (2020) found a prevalence of 10.6% in a similar population. Selçuk et al. (2021) has reported the prevalence rate of 28.5% of PTS in Turkey. The estimates provided by recent reviews are 48.0% (Ma et al., 2021) and 7–53.8% (Xiong et al., 2020). We did not find any study estimating the prevalence of PTS in the Indian children and adolescents in the aftermath of COVID-19. The only available estimate is for adults reporting a prevalence of 28.2% (Singh and Khokhar, 2021). Our findings (68.9%) far exceed these reported prevalence rates, necessitating immediate attention to the mental healthcare for this vulnerable segment of the population. Besides, it also shows the silver lining as a good percentage (39.79%) of those with PTS were also found with PTG.

On analyzing the effect of the demographic variables, namely, age (children and adolescents), gender (men and women), location (urban and rural), family in terms of having siblings and grandparents, it was found that participants differed on intrusion and PTS only in terms of their location of stay with those living in rural areas experiencing significantly higher intrusion and PTS. The reason for this particular difference might be that as compared with urban areas, rural areas lacked the necessary infrastructure to deal with the COVID-19 situation. The availability of hospitals, doctors, and advanced healthcare facilities (oxygen cylinders, PPE kit, masks, sanitizers, etc.) was mostly restricted to the urban areas. In many parts, it was observed that people from rural areas had to travel to urban areas for seeking advanced medical care. This could have led to more intrusive thoughts and greater PTS among them. Although previous studies have reported that women have higher prevalence of PTSD than men (Pyari et al., 2012), we do not find gender difference in this study. It seems that the pandemic affected everyone alike.

The findings also show that those who witnessed hospitalization due to the COVID-19 pandemic in the family reported high prevalence of PTS than those who did not. There was severe shortage of medications, hospital beds, and oxygen in India during the second wave of COVID-19 in India. The hospitalization had put a great toll on the mental health of the families. The experience of struggling to get medical facilities, dealing with finances, and living in the constant fear of losing their loved ones during the hospitalization period seems to be very traumatic experience for the people. Interestingly, the participants who witnessed death of any family member did not have significant PTS as compared with those who did not. Perhaps, seeing the slow deterioration of health of their loved one during hospitalization phase, along with the knowledge of the worst come scenario of the prevailing pandemic somewhere prepared the participants to reach a level of acceptance and acknowledge their grief process. Hence, the experience of struggling for medical facilities and living in the constant fear of losing a loved one as compared with the actual death of a loved one was probably a more traumatic experience for the participants. Moreover, in the present sample, we only had a few cases where death was observed in the family. This could also be the reason why the difference in the two groups failed to reach significance. The preliminary framework of anticipatory grief consisting of three steps (Lindemann, 1994) is in harmony with this finding. The COVID-19 deaths might have become bearable to the family members by separating themselves from their loved ones even before the actual death and adjusting to a new environment where the deceased was missing. This may even allow the grieving ones to form new bonds and relationships.

This study also finds a significant difference with regard to direct-indirect electronic media exposure, with those exposed to electronic media reporting higher PTS than those who were not. However, no significant difference in PTS was observed for those who had indirect exposure to print media. Thus, children and adolescents engaging in more pandemic-related information on electronic media were more likely to show higher PTS symptoms. These findings are partially in congruence with the previous findings stating that mainstream electronic media exposure to COVID-19 could result in PTS-related symptoms (Thompson et al., 2019; Chao et al., 2020; Holman et al., 2020). The results also support the risk factor model of the PTS response, suggesting that pandemic-related media exposure is a potential risk factor for mental health (Freedy et al., 1992; Ben-Ezra et al., 2008). However, the reason why electronic media and not the print media emerged to have a significant effect is that the electronic media showed live videos of people running to hospitals, mass burials, burning pyres, the crying faces, and other horrific scenes. In contrast, the print media consisted of stories and images but lacked the direct firsthand experience and the intense visuals as was shown in electronic media. Therefore, these intense visuals shown in electronic media might have impacted children and adolescents to a greater extent than reading newspaper reports. To add to this, the continuous coverage of the COVID-19 situation by electronic media as against the one-time available information *via* the print media might also have led people to watch news channels more often and for longer periods than usual. Studies claim that prolonged and uncontrolled media exposure could reinforce rumination and intrusive thoughts, activate fear circuitry (Bhushan and Kumar, 2009; Bourne et al., 2013; Holman et al., 2014), and enhance autonomic activation, thus affecting physiological systems (Watkins, 2008; Gerin et al., 2012), leading to the increase in stress. Therefore, the children and adolescents with more electronic media exposure were found with PTS.

Although around 40% of children and adolescents suffering from PTS reported coexistence of PTG, we observed no significant difference in PTG with regard to direct exposure to trauma. However, in the case of indirect exposure to trauma, children and adolescents who were exposed to electronic media were found to report more PTG than those who were not exposed to electronic media. These findings are in congruence with Yoshida et al. (2016), who also observed significantly higher PTG in children who watched the media reports of the Great East Japan Earthquake than those who did not. Studies suggest that watching media coverage of disasters can lead to deliberate rumination, which has been known to facilitate PTG (Taku et al., 2009; Zhou and Wu, 2016; Kim and Bae, 2019). A study by Taku et al. (2009) among US and Japanese samples reported that deliberate rumination strongly predicted the PTG for both the groups. A longitudinal study in the aftermath of an earthquake among the Chinese adolescents suggests that deliberate rumination is conducive to rebuilding the post-traumatic world and eventually elicits PTG after the trauma (Zhou and Wu, 2016). The deliberate rumination is, thus, an important factor facilitating PTG.

We reiterate that having around 40% of the children and adolescents reporting high level of PTS and also experiencing PTG proves the coexistence of PTS and PTG in COVID-affected population as well. This has been reported by other researchers as well (Wang et al., 2018; Fino et al., 2021). We did not directly investigate the possible factors explaining this relationship, but other researchers have reported resilience, emotion regulation, and high social support as mediating factors affecting this coexistence. However, the very fact that the participants of this study with at least one or more siblings experienced higher PTG suggests the significance of high social support from siblings in establishing and/or maintaining this coexistence.

Considering the severe global impact of COVID-19, the missing focus on children and adolescents, and the urban-centric view toward the whole situation, this study adds value to the ongoing research in this field. Very few studies have analyzed the effect of direct and indirect exposure on children and adolescent population. In the Indian context, we found no studies reporting PTS of children and adolescents, and thus, this study fills a very important gap in the existing literature. Moreover, the significant impact of indirect exposure with electronic media contributing significantly to PTS is an important finding bearing serious implications.

However, this study has several limitations. Although the Cronbach’s alpha for the PTS scale was 0.78, the Cronbach’s alpha for the subscales were 0.613, 0.60, and 0.612, respectively. The acceptable range is 0.7 ≤ α < 0.8, and therefore, the PTS level estimated using this tool is also acceptable. Statisticians consider the internal consistency questionable when 0.6 ≤ α < 0.7. Internal consistency suggests the coherence of the items. Thus, from a conservative point of view, one may question the dominance of arousal as a PTS characteristic reported in this study. This is a methodological limitation. The findings of this study are based on the cross-sectional data with a skewed sample in terms of age of the participants. Only 3.7% of participants were children, while the remaining 96.3% were adolescents. This limits the generalization of the findings for the child population. The lack of pre-COVID-19 prevalence rate of PTS limits the findings to the impact of the examined stressful situation only. Furthermore, the data were collected after 30 days of the catastrophic second wave. A longitudinal data would have helped see the unfolding of aftereffect of the pandemic.

## Conclusion

This study shows the repercussions of direct and indirect exposure of COVID-19-related experiences of children and adolescents and its subsequent relationship with PTS and PTG. A substantial percentage of children and adolescents exhibited higher levels of PTS due to COVID-19. The direct exposure to hospitalization and indirect exposure *via* electronic media were significantly associated with PTS. The indirect exposure through print media, however, did not lead to significant rise in PTS. The indirect exposure to electronic media was found to influence COVID-19-related PTS in a major way. Moreover, those who reported PTS also reported PTG, indicating that they probably coexist.

The study holds several important implications. It found arousal to be the most frequently associated characteristics of trauma. Furthermore, children and adolescents of rural areas experienced significantly higher intrusion. These findings can be of immense help in planning mental health interventions. Children and adolescents with at least one or more siblings experienced higher PTG. This finding may hold a utilitarian value in designing the targeted interventions for this vulnerable population. Combining the family and sibling support system with psychological interventions might enable their optimization. The findings have some takeaways for the public health policy. It highlights the positive role of family structures, and the adverse impact of excessive exposure through mass media. An important takeaway is the management of media exposure for better control and choice of the content so as to avoid inadvertent mental health issues.

## Data Availability Statement

The raw data supporting the conclusions of this article will be made available by the authors, without undue reservation.

## Ethics Statement

The studies involving human participants were reviewed and approved by the Institute Ethics Committee, Indian Institute of Technology Kanpur, India. Written informed consent to participate in this study was provided by the participants’ legal guardian/next of kin.

## Author Contributions

BB, SB, and UG were involved in planning the study and data collection and analysis. All authors agreed to be accountable for the content of the study and equally contributed to the planning and conducting of the study.

## Conflict of Interest

The authors declare that the research was conducted in the absence of any commercial or financial relationships that could be construed as a potential conflict of interest.

## Publisher’s Note

All claims expressed in this article are solely those of the authors and do not necessarily represent those of their affiliated organizations, or those of the publisher, the editors and the reviewers. Any product that may be evaluated in this article, or claim that may be made by its manufacturer, is not guaranteed or endorsed by the publisher.

## References

[B1] BBC (2021). *India Coronavirus: New Record Deaths as Virus Engulfs India.* Available online at: https://www.bbc.com/news/world-asia-india-56961940 (accessed May 2, 2021).

[B2] Ben-Ezra M. Palgi Y. Essar N. Sofer H. Haber Y. (2008). Acute stress symptoms, dissociation, and depression among rescue personnel 24 hours after the Bet-Yehoshua train crash: the effects of exposure to dead bodies. *Prehosp. Disaster Med.* 23 461–465. 10.1017/S1049023X00006208 19189616

[B3] Bhushan B. Kumar J. S. (2007). Emotional distress and posttraumatic stress in children surviving the 2004 tsunami. *J. Loss Trauma* 12 245–257. 10.1080/15325020600945996

[B4] Bhushan B. Kumar J. S. (2012). A study of posttraumatic stress and growth in tsunami relief volunteers. *J. Loss Trauma* 17 113–124. 10.1080/15325024.2011.635580

[B5] Bhushan B. Kumar J. S. (2009). Emotional distress and posttraumatic stress in children: the impact of direct versus indirect exposure. *J. Loss Trauma* 14 35–45. 10.1080/15325020802537047

[B6] Bourne C. Mackay C. E. Holmes E. A. (2013). The neural basis of flashback formation: the impact of viewing trauma. *Psychol. Med.* 43 1521–1532. 10.1017/S0033291712002358 23171530 PMC3806039

[B7] Calhoun L. G. Tedeschi R. G. (2006). “The foundations of posttraumatic growth: an expanded framework,” in *Handbook of Posttraumatic Growth: Research & Practice*, eds Calhoun L. G. Tedeschi R. G. (Mahwah, NJ: Lawrence Erlbaum Associates Publishers), 3–23. 10.1371/journal.pone.0214678

[B8] Chao M. Xue D. Liu T. Yang H. Hall B. J. (2020). Media use and acute psychological outcomes during COVID-19 outbreak in China. *J. Anxiety Disord.* 74:102248. 10.1016/j.janxdis.2020.102248 32505918 PMC7255752

[B9] Chen Y. Zhu Z. Lei F. Lei S. Chen J. (2021). Prevalence and risk factors of post-traumatic stress disorder symptoms in students aged 8–18 in Wuhan, China 6 months after the control of COVID-19. *Front. Psychol.* 4651:40575. 10.3389/fpsyg.2021.740575 34721214 PMC8548756

[B10] Cui P. Wang P. Wang K. Ping Z. Wang P. Chen C. (2021). Post-traumatic growth and influencing factors among frontline nurses fighting against COVID-19. *Occup. Environ. Med.* 78 129–135. 10.1136/oemed-2020-106540 33060188

[B11] Fino E. Bonfrate I. Fino V. Bocus P. Russo P. M. Mazzetti M. (2021). Harnessing distress to boost growth in frontline healthcare workers during COVID-19 pandemic: the protective role of resilience, emotion regulation and social support. *Psychol. Med.* 10 1–3. 10.1017/S0033291721000519 33565390 PMC7900668

[B12] Freedy J. R. Resnick H. S. Kilpatrick D. G. (1992). “Conceptual framework for evaluating disaster impact: implications for clinical intervention,” in *Responding to Disaster: A Guide for Mental Health Professionals*, ed. Austin L. S. (American Psychiatric Press), 6–14.

[B13] Gerin W. Zawadzki M. J. Brosschot J. F. Thayer J. F. Christenfeld N. J. Campbell T. S. (2012). Rumination as a mediator of chronic stress effects on hypertension: a causal model. *Int. J. Hypertens.* 2012:453465. 10.1155/2012/453465 22518285 PMC3296188

[B14] Golberstein E. Wen H. Miller B. F. (2020). Coronavirus disease 2019 (COVID-19) and mental health for children and adolescents. *JAMA Pediatr.* 174 819–820. 10.1001/jamapediatrics.2020.1456 32286618

[B15] Hamam A. A. Milo S. Mor I. Shaked E. Eliav A. S. Lahav Y. (2021). Peritraumatic reactions during the COVID-19 pandemic– The contribution of posttraumatic growth attributed to prior trauma. *J. Psychiatr. Res.* 132 23–31. 10.1016/j.jpsychires.2020.09.029 33038562 PMC7525333

[B16] Holman E. A. Garfin D. R. Silver R. C. (2014). Media’s role in broadcasting acute stress following the Boston Marathon bombings. *Proc. Natl. Acad. Sci. U.S.A.* 111 93–98. 10.1073/pnas.1316265110 24324161 PMC3890785

[B17] Holman E. A. Garfin D. R. Lubens P. Silver R. C. (2020). Media exposure to collective trauma, mental health, and functioning: does it matter what you see? *Clin. Psychol. Sci.* 8 111–124. 10.1177/2167702619858300

[B18] Holmes E. A. O’Connor R. C. Perry V. H. Tracey I. Wessely S. Arseneault L. (2020). Multidisciplinary research priorities for the COVID-19 pandemic: a call for action for mental health science. *Lancet Psychiatry* 7 547–560. 10.1016/S2215-0366(20)30168-1 32304649 PMC7159850

[B19] Horowitz M. Wilner N. Alvarez W. (1979). Impact of event scale: a measure of subjective stress. *Psychosom. Med.* 41 209–218. 10.1097/00006842-197905000-00004 472086

[B20] Hou T. Y. Mao X. F. Dong W. Cai W. P. Deng G. H. (2020). Prevalence of and factors associated with mental health problems and suicidality among senior high school students in rural China during the COVID-19 outbreak. *Asian J. Psychiatry* 54 102305. 10.1016/j.ajp.2020.102305 32668388 PMC7348589

[B21] Hyland P. Shevlin M. Murphy J. McBride O. Fox R. Bondjers K. (2021). A longitudinal assessment of depression and anxiety in the Republic of Ireland before and during the COVID-19 pandemic. *Psychiatry Res.* 300:113905. 10.1016/j.psychres.2021.113905 33827013 PMC9755108

[B22] Jain V. K. Iyengar K. P. Vaishya R. (2021). Differences between First wave and Second wave of COVID-19 in India. *Diabetes Metab. Syndr.* 15 1047–1048. 10.1016/j.dsx.2021.05.009 33992554 PMC8106236

[B23] Joseph S. Williams R. (2005). Understanding posttraumatic stress: theory, reflections, context and future. *Behav. Cogn. Psychother.* 33 423–441. 10.1017/S1352465805002328

[B24] Joshi R. K. Mehendale S. M. (2021). Prevention and control of COVID-19 in India: strategies and options. *Med. J. Armed Forces India* 77:S237. 10.1016/j.mjafi.2021.05.009 34334886 PMC8313043

[B25] Kilmer R. P. Gil-Rivas V. Tedeschi R. G. Cann A. Calhoun L. G. Buchanan T. (2009). Use of the revised posttraumatic growth inventory for children. *J. Traumat. Stress* 22 248–253. 10.1002/jts.20410 19462437 PMC2827205

[B26] Kim E. Bae S. (2019). Gratitude moderates the mediating effect of deliberate rumination on the relationship between intrusive rumination and post-traumatic growth. *Front. Psychol.* 10:2665. 10.3389/fpsyg.2019.02665 31849774 PMC6901784

[B27] Lindemann E. (1994). Symptomatology and management of acute grief. *Am. J. Psychiatry* 151(Suppl. 6), 155–160. 10.1176/ajp.151.6.155 8192191

[B28] Ma L. Mazidi M. Li K. Li Y. Chen S. Kirwan R. (2021). Prevalence of mental health problems among children and adolescents during the COVID-19 pandemic: a systematic review and meta-analysis. *J. Affect. Disord.* 293 78–89. 10.1016/j.jad.2021.06.021 34174475 PMC9711885

[B29] Mertens G. Gerritsen L. Duijndam S. Salemink E. Engelhard I. M. (2020). Fear of the coronavirus (COVID-19): predictors in an online study conducted in March 2020. *J. Anxiety Disord.* 74:102258. 10.1016/j.janxdis.2020.102258 32569905 PMC7286280

[B30] Meyers L. S. Gamst G. Guarino A. J. (2016). *Applied Multivariate Research: Design and Interpretation.* San Diego, CA: Sage publications.

[B31] Ministry of Home Affairs (2020). *Lockdown Measures for Containment of COVID-19 Pandemic in the Country to Continue to Remain in Force up to May 3, 2020.* Available online at: https://pib.gov.in/PressReleasePage.aspx?PRID=1614481 (accessed May 3, 2020).

[B32] Nader K. O. Pynoos R. S. Fairbanks L. A. Al-Ajeel M. Al-Asfour A. (1993). A preliminary study of PTSD and grief among the children of Kuwait following the Gulf crisis. *Br. J. Clin. Psychol.* 32 407–416. 10.1111/j.2044-8260.1993.tb01075.x 8298537

[B33] Perrin S. Meiser-Stedman R. Smith P. (2005). The Children’s Revised Impact of Event Scale (CRIES): validity as a screening instrument for PTSD. *Behav. Cogn. Psychother.* 33 487–498. 10.1017/S1352465805002419

[B34] Pfefferbaum B. Nixon S. J. Tivis R. D. Doughty D. E. Pynoos R. S. Gurwitch R. H. (2001). Television exposure in children after a terrorist incident. *Psychiatry* 64 202–211. 10.1521/psyc.64.3.202.18462 11708044

[B35] Pyari T. T. Kutty R. V. Sarma P. S. (2012). Risk factors of post-traumatic stress disorder in tsunami survivors of Kanyakumari District, Tamil Nadu, India. *Indian J. Psychiatry* 54 48–53. 10.4103/0019-5545.94645 22556437 PMC3339219

[B36] Safi M. (2021). *India’s Shocking Surge in Covid Cases follows Baffling Decline. The Guardian.* Available online at: https://www.theguardian.com/world/2021/apr/21/india-shocking-surge-in-covid-cases-follows-baffling-decline (accessed April 21, 2021).

[B37] Selçuk E. B. Demir A. Ç Erbay L. G. Özcan ÖÖ Gürer H. Dönmez Y. E. (2021). Anxiety, depression and post-traumatic stress disorder symptoms in adolescents during the COVID-19 outbreak and associated factors. *Int. J. Clin. Pract.* 75:e14880. 10.1111/ijcp.14880 34528350 PMC8646813

[B38] Singh S. P. Khokhar A. (2021). Prevalence of posttraumatic stress disorder and depression in general population in India during COVID-19 pandemic home quarantine. *Asia Pac. J. Public Health* 33 154–156. 10.1177/1010539520968455 33198479

[B39] Stallard P. Velleman R. Baldwin S. (1999). Psychological screening of children for posttraumatic stress disorder. *J. Child Psychol. Psychiatry* 40 1075–1082. 10.1111/1469-7610.00525 10576537

[B40] Taku K. Cann A. Tedeschi R. G. Calhoun L. G. (2009). Intrusive versus deliberate rumination in posttraumatic growth across US and Japanese samples. *Anxiety Stress Coping* 22 129–136. 10.1080/10615800802317841 18937084

[B41] Tan W. Hao F. McIntyre R. S. Jiang L. Jiang X. Zhang L. (2020). Is returning to work during the COVID-19 pandemic stressful? A study on immediate mental health status and psychoneuroimmunity prevention measures of Chinese workforce. *Brain Behav. Immunity* 87 84–92. 10.1016/j.bbi.2020.04.055 32335200 PMC7179503

[B42] Tedeschi R. G. Calhoun L. G. (1995). *Trauma and Transformation.* San Diego, CA: Sage.

[B43] Tedeschi R. G. Calhoun L. G. (1996). The posttraumatic growth inventory: measuring the positive legacy of trauma. *J. Traumat. Stress* 9 455–471. 10.1002/jts.2490090305 8827649

[B44] Thompson R. R. Jones N. M. Holman E. A. Silver R. C. (2019). Media exposure to mass violence events can fuel a cycle of distress. *Sci. Adv.* 5:eaav3502. 10.1126/sciadv.aav3502 31001584 PMC6469939

[B45] Tomaszek K. Muchacka-Cymerman A. (2020). Thinking about my existence during COVID-19, I feel anxiety and awe— The mediating role of existential anxiety and life satisfaction on the relationship between PTSD symptoms and post-traumatic growth. *Int. J. Environ. Res. Public Health* 17:7062. 10.3390/ijerph17197062 32992515 PMC7579162

[B46] Vindegaard N. Benros M. E. (2020). COVID-19 pandemic and mental health consequences: systematic review of the current evidence. *Brain Behav. Immunity* 89 531–542. 10.1016/j.bbi.2020.05.048 32485289 PMC7260522

[B47] Wang W. Wu X. Tian Y. (2018). Mediating roles of gratitude and social support in the relation between survivor guilt and posttraumatic stress disorder, posttraumatic growth among adolescents after the Ya’an earthquake. *Front. Psychol.* 9:2131. 10.3389/fpsyg.2018.02131 30455660 PMC6230928

[B48] Watkins E. R. (2008). Constructive and unconstructive repetitive thought. *Psychol. Bull.* 134 163–206. 10.1037/0033-2909.134.2.163 18298268 PMC2672052

[B49] World Health Organization [WHO] (2020). *WHO Director-General’s Opening Remarks at the Media Briefing on COVID-19–11 March 2020.* Available online at: https://www.who.int/director-general/speeches/detail/who-director-general-s-opening-remarks-at-the-media-briefing-on-covid-19—11-march-2020 (accessed March, 2020).

[B50] Xiao S. Luo D. Xiao Y. (2020). Survivors of COVID-19 are at high risk of posttraumatic stress disorder. *Global Health Res. Policy* 5:29. 10.1186/s41256-020-00155-2 32514428 PMC7273810

[B51] Xiong J. Lipsitz O. Nasri F. Lui L. M. Gill H. Phan L. (2020). Impact of COVID-19 pandemic on mental health in the general population: a systematic review. *J. Affect. Disord.* 277 55–64. 10.1016/j.jad.2020.08.001 32799105 PMC7413844

[B52] Yeung N. C. Lau J. T. Yu N. X. Zhang J. Xu Z. Choi K. C. (2018). Media exposure related to the 2008 Sichuan earthquake predicted probable PTSD among Chinese adolescents in Kunming, China: a longitudinal study. *Psychol. Trauma* 10:253. 10.1037/tra0000121 27428553

[B53] Yoshida H. Kobayashi N. Honda N. Matsuoka H. Yamaguchi T. Homma H. (2016). Post-traumatic growth of children affected by the Great East Japan Earthquake and their attitudes to memorial services and media coverage. *Psychiatry Clin. Neurosci.* 70 193–201. 10.1111/pcn.12379 26821650

[B54] Yule W. (1998). “Anxiety, depression, and posttraumatic stress disorder in children,” in *The NFER Child Portfolio*, ed. Sclare I. (Windsor, ON: NFER-Nelson).

[B55] Zarocostas J. (2020). How to fight an infodemic. *Lancet* 395:676. 10.1016/S0140-6736(20)30461-X 32113495 PMC7133615

[B56] Zhang Y. Zhuang L. Y. Yang W. W. (2020). Survey of symptoms of post-traumatic stress disorder in middle school students during the COVID-19 pandemic: taking Chengdu Shude Middle School as an example. *Educ. Sci. Forum* 17 45–48.

[B57] Zhou X. Wu X. (2016). The relationship between rumination, posttraumatic stress disorder, and posttraumatic growth among Chinese adolescents after earthquake: a longitudinal study. *J. Affect. Disord0* 193 242–248. 10.1016/j.jad.2015.12.076 26773915

